# Bibliographical

**Published:** 1882-10

**Authors:** 


### BIBLIOGRAPHICAL.

*Lascerations of The Female Perineum and Visigo-Vaginal Fistulae. Their History and Treatment, by D. Hayes Agnew, M. D. Published by P. Blakiston, Son & Co., Philadelphia. Paper,* 75 *cents.*

From Dr. Agnew's large experience he gives in this present work a valuable contribution to medical literature. A history and treatment of difficult cases is given, as well as illustrations of parts treated and drawings of instruments used. The author's reputation as authority and ability needs no commendation by us.

*The Change in Life in Health and Disease. By Edward John Tilt, M. D. Published by P. Blakiston, Son & Co., Philadelphia. Paper,* 75 *cents; cloth,* $1.25.

Dr. Tilt's clear, forcible and concise style has gained for him a reputation as a medical writer that is enviable, and the issuing of this new edition of a clinical treatise on the diseases of the Ganglionis Nervous system incidental to women at the decline of life, is timely and considerate.

*On Slight Ailments: Their Nature and Treatment. By Lionel S. Beale, M. D., F.R.S. Published by P. Blakiston, Son & Co., Philadelphia. Cloth.*

With the appearance of this book comes the announcement that the new edition is published simultaneously with the London edition. Quite a number of alterations have been made in the text; an index is given, as well as an epitome of new cases that have lately come under the author's observation. The style is lucid, and written for the express purpose of giving decided and clear information to the student, as well as the physician, in active practice. These observations cover all the " slight ailments that flesh is heir to," and offer some sound advice about their instantaneous treatment, and handles imposition and vulgarity with severity.

*Lindsay & Blackiston's Physicians' Visiting List*, has put in its annual visit for 1883, in its usual popular style. It is handsome, neat, concise, and nothing better need be looked for by the physscian. Philadelphia : P. Blakiston, Son & Co.

*The Multum in Parvo Reference and Dose Book. By C. Henri Leonard, M. A., M. D. Published by the Ill'd Medical Journal Co., Detroit. Paper*, 30 *cents. Cloth*, 75 *cents.*

A neat, handy, concise and valuable little work, giving full suggestions as to administration, prescribing and preparation of drugs.

Pamphlets Received.—We are sorry that we cannot spare more space for a review of the pamphlets which we have received, many of which are truly valuable, and form a collection of interesting and instructive reading. To the authors we return thanks for their kind remembrance, and we call the attention of our readers to the following list: "Ten Years' Experience in the Treatment of Stricture of the Urethra by Electrolysis," "A Successful Ovariotomy in 1866," "Stricture of the Rectum Treated by Electrolysis," by Dr. Robert Newman. "The Prescription of Proprietary Medicine for the Sick, its Demoralizing Effects upon the Medical Profession," an essay read before the Connecticut Medical Society, by C. A. Lindsley, M. D. "Report on Ophthalmology," made to the Medical and Chirugical Faculty of Maryland by Julian J. Chisolm, M. D. "Diptheritic Ulceration of the Air-passages and its relation to Pulmonary Pthisis," by John N. Mackenzie, M. D. "Note on the Microscopic Appearances Presented by the Blood of Scarlatina and Typhoid Fever," by J. H. Kidder, Surgeon U. S. Navy. "The Female Perineum," by T. G. Comstock, M. D. "The Application of Premull in Diseases of the Uterus, Ovaries and Peri-Uterine Structures," by V. V. Taliaferro, M. D. "The Genius of Medicine," the annual address presented to the Florida State Medical Association at Pensacola, by Robt. B. S. Hargis, M. D. "Abortive Treatment of Mammary Abscesses and The Cure of Fissured Nipples by Means of a New and Effectual Compress," by Geo. H. Noble, M. D. "Treatment of Consumption Indicated by the Discoveries of Koch and others of its Parasitic Origin," by M. L. James, M. D. "A Study of Rupture of the Bladder," with some explanations of experiments bearing upon the closure of the vesical wound, by Alex. W. Stein, M. D. This is quite an extensive monograph and deals in an entertaining manner with the pathology, symtomatology, diagnosis and treatment of ruptures of the bladder. It is published by Gustav E. Stechert of this city. The last of these essays is a "Contribution to the subject of Nerve Stretching," by William J. Morton, M. D., reprinted from the *Journal of Nervous and Mental Diseases.*

*The November Century*, which is the first number of a new volume, gives promise of even increased excellence for the magazine during its second year under the new name. Pictorially the November number shows that the *Century* is as ambitious as ever for the reputation of American wood-engraving, as witness the frontispiece portrait of Florence Nightingale and the full-page portrait of Henry James, Jr., both by Cole; Elbridge Kingsley's beautiful full-page engraving, direct from nature, of a view in New England woods (accompanying which is a description by the engraver of his manner of working); the full-page reproduction, by Kruell, of the ideal bronze head which is one of the costly art-treasures of the British Museum; Mary Hallock Foote's refined and charming illustrations, engraved by Miss Powell and by Cole; and the many other pictures by well-known artists, some of which have a special interest as the exponents of a new process of art reproduction.

Though the art side of the November *Ceutury* is so conspicuous, the contents offer striking proof of a tendency to make the literary side of the magazine paramount and of the greatest possible excellence and importance in travel, biography, fiction, poetry, criticism, and in the discussion of the foremost public questions.

*The Popular Science Monthly* for November opens with a most valuable article on the important subject of "Sewer-Gas," by DR. FRANK H. HAMILTON. It is an unsettled and most perplexing problem, physicians and architects and sanitary engineers being much at war about it, yet it can not be neglected because of their disagreements. Dr. Hamilton sums up the subject clearly and judicially, and, what is more, indicates the only safe ground to be taken in regard to it. The second article, by Professor Du Bois-Reymond, on "The Science of the Present Period," is a masterly vindication of its influence and its broader tendencies. Dr. Nathan Allen gives the latest form to "The Law of Human Increase," and draws from it certain practical conclusions of very great importance. "Science in Relation to the Arts," by Dr. Siemens, is the address of the President of the British Association for the Advancement of Science, and is especially instructive in its discussion of electric and gas lighting. Dr. Oswald's second paper on "Physiognomic Curiosities" is in his best vein, bright, crisp, witty, and full of curious information, as new as it is interesting. The article on "Scientific Farming at Rothamsted," by Dr. Manly Miles, is a most timely and well-digested account of the systematic agricultural experiments of Lawes and Gilbert, which are of far greater value, both theoretical and practical, than is generally supposed. Dr. Miles's paper is of capital interest. The original and striking paper of that clear and excellent writer Professor Grant Allen, entitled "Who was Primitive Man?" appears without abridgment in the November "Monthly." It goes far toward clearing away some tenacious difficulties of a perplexing subject. "Some Curious Vegetable Growths," by W. H. Larrabee, "The British Lion," by W. Boyd Dawkins. "Life among the Battas of Sumatra," by Dr. Schreiber, are also very readable articles. There are a biography and portrait of the French chemist, Wurtz, and the usual mass of miscellaneous information at the close, which is always read first in *The Popular Science Monthly*.

*The Journal of Cutaneous and Venereal Diseases*, a new magazine, displays in its first issue considerable ability and judgment in the selection of subjects and editorial management. It fills a prominent place in medical literature, and opens a new medium for special discussion. It is handsomely illustrated by a colored frontispiece.

